# Genetic structure of dinoflagellate symbionts in coral recruits differs from that of parental or local adults

**DOI:** 10.1002/ece3.9312

**Published:** 2022-09-19

**Authors:** Mary Alice Coffroth, Noel J. Leigh, Shelby E. McIlroy, Margaret W. Miller, H. David Sheets

**Affiliations:** ^1^ Department of Geology University at Buffalo Buffalo New York USA; ^2^ Graduate Program in Evolution, Ecology and Behavior University at Buffalo Buffalo New York USA; ^3^ NOAA Southeast Fisheries Science Center Miami Florida USA; ^4^ Graduate Program in Data Analytics Canisius College Buffalo New York USA; ^5^ Present address: School of Biological Sciences, The Swire Institute of Marine Science The University of Hong Kong Hong Kong China; ^6^ Present address: SECORE International, Inc. Miami Florida USA

**Keywords:** coral, mutualism, onset of symbiosis, population structure, Symbiodiniaceae, zooxanthellae

## Abstract

The symbiotic relationship between dinoflagellate algae in the family Symbiodiniaceae and scleractinian corals forms the base of the tropical reef ecosystem. In scleractinian corals, recruits acquire symbionts either “vertically” from the maternal colony or initially lack symbionts and acquire them “horizontally” from the environment. Regardless of the mode of acquisition, coral species and individual colonies harbor only a subset of the highly diverse complex of species/taxa within the Symbiodiniaceae. This suggests a genetic basis for specificity, but local environmental conditions and/or symbiont availability may also play a role in determining which symbionts within the Symbiodiniaceae are initially taken up by the host. To address the relative importance of genetic and environmental drivers of symbiont uptake/establishment, we examined the acquisition of these dinoflagellate symbionts in one to three‐month‐old recruits of *Orbicella faveolata* to compare symbiont types present in recruits to those of parental populations versus co‐occurring adults in their destination reef. Variation in chloroplast 23S ribosomal DNA and in three polymorphic microsatellite loci was examined. We found that, in general, symbiont communities within adult colonies differed between reefs, suggesting that endemism is common among symbiont populations of *O. faveolata* on a local scale. Among recruits, initial symbiont acquisition was selective. *O. faveolata* recruits only acquired a subset of locally available symbionts, and these generally did not reflect symbiont populations in adults at either the parental or the outplant reef. Instead, symbiont communities within new recruits at a given outplant site and region tended to be similar to each other, regardless of parental source population. These results suggest temporal variation in the local symbiont source pool, although other possible drivers behind the distinct difference between symbionts within *O. faveolata* adults and new generations of recruits may include different ontogenetic requirements and/or reduced host selectivity in early ontogeny.

## Graphical abstract

Although showing fidelity at genus/species level, at the level of genotype/population, symbiont populations within recruits did not reflect the symbiont populations in adults at either the parental or the outplant reef. Instead, symbiont communities within new recruits at a given outplant site tended to be similar to each other, regardless of parental source population. While drivers behind the distinct difference between symbionts within *O. faveolata* adults and new generations of recruits may include different ontogenetic requirements and/or reduced host selectivity in early ontogeny, our results indicate that variation in the local symbiont source pool likely explains our observations. This change in the local symbiont pools may reflect adaptations in symbionts in response a changing climate, thus suggesting the possibility for a new generation of more resilient corals.

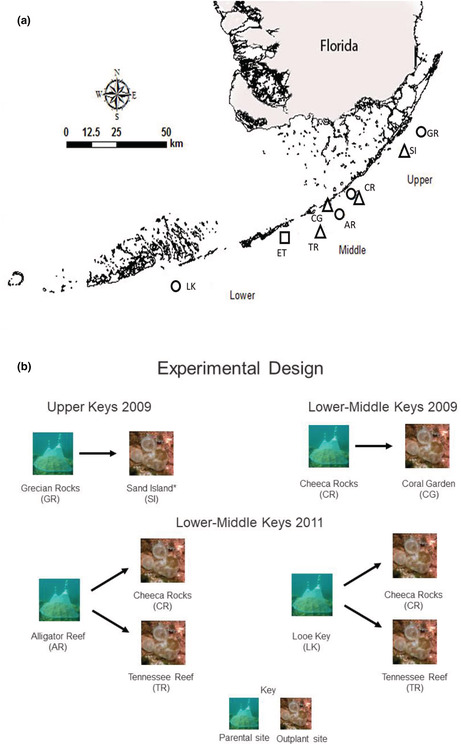

## 1 INTRODUCTION

Reef corals form a symbiosis with unicellular dinoflagellates in the family Symbiodiniaceae providing the foundation for one of the most biodiverse and economically important ecosystems on earth (Moberg & Folke, 1999; Muscatine & Porter, 1977; Reaka‐Kudla, 1997). The importance of this symbiosis cannot be overstated as it is crucial to the survival and development of reefs found around the world. Coral reefs are threatened by numerous anthropogenic perturbations (Smith & Buddemeier, 1992), and while certain aspects of this important mutualism are well known, many questions of the establishment and maintenance of this mutualism remain.

These algal symbionts, originally classified as a single species (*Symbiodinium*), are now recognized as a diverse family (Symbiodiniaceae) made up of at least 11 genera (LaJeunesse et al., 2018, 2021; Nitschke et al., 2020; Pochon & LaJeunesse, 2021) with a range of physiological characteristics (McIlroy et al., 2016; Takahashi et al., 2009; Warner et al., 1999). Corals acquire symbionts via two different methods, vertical and horizontal transmission (Baird et al., 2009). In coral species with vertical transmission, the symbiont is passed directly from the maternal colony to the offspring, whereas in species with horizontal transmission, the dominant mode for most coral taxa, the offspring acquires its symbionts from the environmental source pool.

In host species with horizontal transmission, numerous studies have demonstrated that newly settled recruits can initially acquire multiple symbiont types (Coffroth et al., 2001; Cumbo et al., 2013; Gómez‐Cabrera et al., 2008; Little et al., 2004; Poland et al., 2013; Yamashita et al., 2013). These often differ from the predominant symbiont species in the adult host, but over time symbiont diversity is reduced to the single (in most cases) symbiont species that dominates in the adult symbiosis, suggesting a level of specificity (Abrego et al., 2009a; Goulet, 2006; LaJeunesse, 2002; LaJeunesse et al., 2010; Little et al., 2004; Poland & Coffroth, 2017; Rodriguez‐Lanetty et al., 2006; Thornhill et al., 2014).

The factors that govern the symbiont type acquired initially and whether patterns of initial acquisition vary at the population level remain unclear. Poland and Coffroth (2017) demonstrated that in the octocoral *Briareum asbestinum*, which has horizontal symbiont transmission, recruits, raised in a common location, acquired symbiont genotypes unique to the parental population. Quigley, Bay, and Willis (2017) and Quigley, Willis, and Bay (2017) also found that in host with horizontal transmission, host genetics accounted for 29% of variation in symbiont communities. This suggests that there may be an inherited genetic predisposition that influences initial symbiont acquisition at the population level in at least some cnidarians. However, in many cases symbiont communities within newly settled cnidarian recruits differ from nearby adults (Abrego et al., 2009b; Andras et al., 2011; Gómez‐Cabrera et al., 2008; Little et al., 2004; Mellas et al., 2014; Poland et al., 2013; Thornhill, Daniel, et al., 2006; Thornhill, LaJeunesse, et al., 2006). For example, Gómez‐Cabrera et al. (2008) reported that new recruits of *Acropora longicyathus* were dominated by symbionts within the genus *Symbiodinium* (formerly Clade A) while the majority of neighboring adults harbored symbionts within *Cladocopium* (formerly Clade C). They found no effect of parentage or location within the reef and suggest that the ontogenetic change observed in symbiont type between recruits and adults was due to host selection or differing microhabitats of recruit and adult. Even within the octocoral *B. asbestinum*, which displays this inherited predisposition to a single symbiont type over time, newly settled recruits initially host multiple symbiont types (Poland et al., 2013). Thus, in some cases, initial symbiont acquisition may not reflect a host specificity but be due to other factors such as ontogenetic requirements, local symbiont source pool, environmental conditions, immature host immune system, highly infectious types and/or symbiont competition (Abrego et al., 2009b, 2012; Chan et al., 2019; Fitt, 1985; Hawkins et al., 2016; McIlroy et al., 2019; McIlroy & Coffroth, 2017; Puill‐Stephan et al., 2012; Quigley et al., 2019; Quigley, Bay, & Willis, 2017; Wilkerson et al., 1988). The contrasting findings of these studies (Abrego et al., 2009b; Poland et al., 2013) point to our lack of understanding of the factors that determine initial symbiont uptake and the final symbiont assemblage in the adult.

In this study, using both markers that distinguish symbiont taxa among species within genus (hypervariable regions of chloroplast 23S rDNA) and within populations (microsatellites), we examined symbiont acquisition of newly settled recruits of the scleractinian coral *Orbicella faveolata* that were outplanted to non‐natal reefs. We compared symbionts in the newly settled recruits to symbionts in adults at both the parental and outplant sites. We used this comparison to address the question: do symbiont communities of recruits resemble those found within the parental populations of their natal reefs (shaped by host genetics), or those within adults at the settlement site (shaped by environmental conditions and/or ambient symbiont source pool)?

Identifying factors that determine the symbiont types acquired by newly settled coral recruits may be crucial for future preservation of coral reefs. As sea surface temperatures rise, there has been an increase in coral bleaching and associated mortality (Heron et al., 2016; Hughes et al., 2018; Oliver et al., 2018; van Hooidonk et al., 2016). It has been proposed that if corals can be induced to take up, shuffle or switch to algal symbiont types which are more resilient to increasing temperatures, coral mortality may be reduced (Baker, 2003; Buddemeier & Fautin, 1993; National Academies of Science, 2018). Therefore, understanding the influence of genetics and environmental factors on symbiont acquisition will aid in understanding the potential for corals to respond to these perturbations.

## 2 MATERIAL AND METHODS

### 2.1 Field methods

#### 2.1.1 Study organism

The model organism for this study was *Orbicella faveolata*, a scleractinian coral found throughout the Caribbean which acquires symbionts via horizontal transmission (Baird et al., 2009; Szmant, 1991). *O. faveolata* can harbor symbiont types within four Symbiodiniaceae genera: *Symbiodinium*, *Breviolum*, *Cladocopium*, and *Durusdinium* (formerly Clades A, B, C, and D, respectively) throughout its life. While symbiont types within each of these genera are found within *O. faveolata* in the Florida Keys, symbiont species within the *Breviolum* B184/B1 type (based on a hypervariable region of domain V of the 23S rDNA chloroplast gene and ITS2) dominate the symbiosis in shallow (5 m) water of the Florida Keys (Baums et al., 2010; LaJeunesse, 2002; Thornhill et al., 2009).

*Orbicella faveolata* is a hermaphroditic broadcast spawning coral that releases egg‐sperm bundles 6–8 days after the full moon in August or September (Szmant et al., 1997). At this time, egg‐sperm bundles float to the surface of the water column where they mix and are fertilized (Sanchez et al., 1999). After 36–48 h, the fertilized embryos have developed into swimming aposymbiotic coral planular larvae (Schwarz et al., 2008). These planulae then settle onto the substrate and develop into a coral polyp after 3–7 days.

#### 2.1.2 Study sites

**FIGURE 1:**
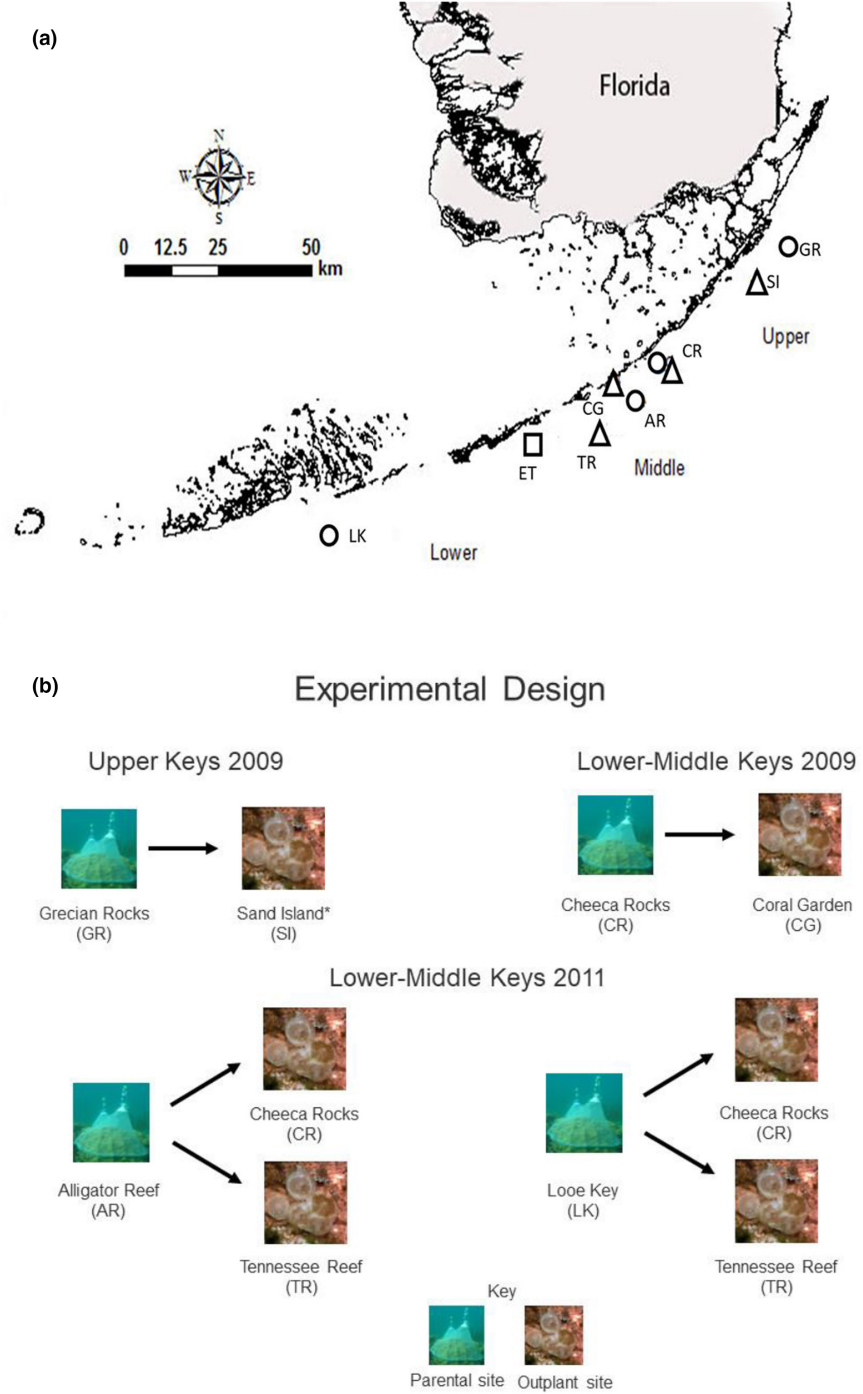
Study sites in the Florida Keys. (a) Map of the Florida Keys indicating spawning (parental) locations (circles), outplant locations (triangles), and tile conditioning site in 2011 (square). Site name, abbreviation, and sampling information are given in Table 1. Map was generated using ArcGIS version 9. (b) Outline of experimental design showing parental site and outplant site for each year.

Adult tissue samples were collected from reefs in the Upper, Middle, and Lower Florida Keys in both deep and shallow sites (Figure 1a, Table 1). The Upper Keys included two shallow sites, SI (Sand Island) and GR (Grecian Rocks; 2 and 6 m, respectively). The Middle Keys included three shallow sites, CG (Coral Gardens), CR (Cheeca Rocks), and ET (East Turtle; 4, 5, and 6 m, respectively) and two deeper sites, TR (Tennessee Reef) and AR (Alligator Reef; 12 and 15 m, respectively). The Lower Keys adult tissue samples were collected at LK (Looe Key; 5 m). For logistical reasons, spawning collections were made at GR, CR, AR, and LK (Figure 1b). After settlement in the laboratory, recruits were outplanted to SI, CG, CR, and TR (Figure 1b). Sites that served as the sources of gametes are referred to as parental sites, and the sites to which the newly settled recruits were transferred are referred to as outplant sites. At the time of the experiments, all sites had large (1–2 m), healthy‐looking *O. faveolata* colonies with little to no evidence of bleaching or disease.

**TABLE 1:** Study site details

Sample code	Type	Parental site (year)	Outplant site (year)	Pre‐conditioning site (year)	Depth	*N*_cp23S_	*N*_microsat_
SI	Adult	NA	Sand Island	NA	2 m	14	14
GR	Adult	Grecian Rocks (2009)	NA	NA	6 m	26	14
CR	Adult	Cheeca Rocks (2009)	Cheeca Rocks (2011)	NA	5 m	35	22
CG	Adult	NA	Coral Garden (2009)	NA	4 m	53	35
TR	Adult	NA	Tennessee Reef (2011)	NA	12 m	27	22
AR	Adult	Alligator Reef (2011)	NA	NA	15 m	29	27
ET	Adult	East Turtle (2011)	NA	NA	6 m	22	14
LK	Adult	Looe Key (2011)	NA	NA	5 m	50	75
GR‐SI	Recruit	Grecian Rocks (2009)	Sand Island (2009)	Sand Island (2009)	2 m	109	24
CR‐CG	Recruit	Cheeca Rocks (2009)	Coral Garden (2009)	Craig Key (2009)	4 m	16	4
AR‐CR	Recruit	Alligator Reef (2011)	Cheeca Rocks (2011)	East Turtle (2011)	5 m	23	6
LK‐CR	Recruit	Looe Key (2011)	Cheeca Rocks (2011)	East Turtle (2011)	5 m	17	6
AR‐TR	Recruit	Alligator Reef (2011)	Tennessee Reef (2011)	East Turtle (2011)	12 m	10	11
LK‐TR	Recruit	Looe Key (2011)	Tennessee Reef (2011)	East Turtle (2011)	12 m	4	4

*Note*: Reef locations and notations used in this study. Parental sites are those adult populations where egg‐sperm bundles were collected. Outplant sites indicate the location where recruits were outplanted. Adult *Orbicella faveolata* populations were sampled at all sites. NA—not applicable. *N*_cp23S_ = number of symbiont samples used in cp‐type analysis, *N*_microsat_ = number of symbiont samples used in microsatellite analysis. Sample size for the microsatellite analysis is smaller than that for the cp‐analysis because of depletion of DNA due to the need to screen multiple loci and repeat analyses to obtain results. (see Material and Methods for details).

#### 2.1.3 Gamete collection, recruit rearing, and outplanting

Egg‐sperm bundles were collected by placing a mesh “tent” over the adult *O. faveolata* colony following the techniques described by Miller (2014). Collection cups with egg‐sperm bundles from the same reef were combined, diluted with filtered seawater (FSW, 1.6 μm), and incubated for 1–2 h to allow fertilization to occur. Excess sperm was then removed through a series of FSW rinses, and developing embryos were placed in FSW. Spawn from each collection site was maintained separately in FSW in the laboratory for a period of 2 weeks to allow settlement and metamorphosis before outplanting to the field. During this time, the water was replaced a minimum of two times a day with FSW, either manually, or continuously with a recirculating system.

Larvae were settled onto terracotta tiles pre‐conditioned either in the field or in FSW in the laboratory. Pre‐conditioning ensured that tiles were coated with a bacterial film and/or crustose coralline algae, which has been found to promote coral settlement (Heyward & Negri, 1999). Here, we refer to larvae that metamorphosed and settled onto tiles in the laboratory as newly settled corals or recruits. Reef pre‐conditioned tiles were placed in the field for at least a month prior to spawning. Larvae from the Upper Keys were reared at a shore‐based site in Key Largo, FL and settled onto tiles pre‐conditioned at SI, the outplant site. All larvae from the Middle and Lower Keys were reared at Keys Marine Laboratory (KML, Long Key, FL) and settled onto pre‐conditioned tiles. In 2009, the tiles were pre‐conditioned at a shallow nearshore hard bottom site (Craig Key, 2–3 m) where *O. faveolata* did not occur. In 2011, tiles were pre‐conditioned in FSW at KML or at ET where *O. faveolata* was one of the dominant corals. Lab pre‐conditioned tiles were placed in FSW for a month to allow a bacterial film to develop. Prior to deployment of the coral recruits in 2011, 2‐week‐old recruits were collected from tiles pre‐conditioned in the field at ET to evaluate whether the newly settled corals had acquired symbionts from these tiles while being maintained in the laboratory (30 settlers per spawning site [AR and LK], 60 total).

When the recruits had metamorphosed and attached to the tiles, they were transported to the appropriate outplant location (Table 1) and attached vertically to a PVC rack approximately 0.2 m above the substrate. Newly settled recruits are referred to by spawning site‐outplant site. For example, larvae spawned at GR and outplanted to SI are referred to GR‐SI recruits and CR‐CG recruits were those spawned at CR and outplanted to CG. The tiles were retrieved 1–3 months after outplanting and recruits were removed from the tiles and preserved individually in 95% ethanol for subsequent molecular analysis (see Table 1 for sample sizes, range 4–109).

#### 2.1.4 Field collection of coral adult tissue

*Orbicella faveolata* adults were sampled at the parental and outplant reefs as well as at the site where settlement tiles were pre‐conditioned in 2011 (Table 1) to identify the suite of symbionts within *O. faveolata* colonies at each site. A single polyp was sampled from the top, middle, and bottom of adult *O. faveolata* colonies using a syringe, and the tissue sample was filtered in situ onto a 13‐mm glass fiber filter (Correa et al., 2009). The 15 to 50 colonies sampled at each site generated a total of 45 to 150 samples per site, which were used to capture the symbiont diversity within *O. faveolata* at a site as a whole. At the parental sites, samples were collected from a combination of the corals that spawned as well as from other nearby conspecific colonies. Filters containing tissue samples were preserved in either a 20% salt‐saturated dimethyl sulfoxide solution (Seutin et al., 1991) or 95% ethanol.

### 2.2 Laboratory methods

#### 2.2.1 Molecular identification of symbionts from *O. faveolata*

DNA was extracted from adult tissue collected on 13‐mm glass fiber filters or tissue from newly settled corals following Coffroth et al. (1992). Extracted DNA was re‐suspended in TE buffer (5–15 μl), diluted to a concentration of approximately 5–10 ng/μl, and used to amplify the appropriate gene region as indicated below. To determine symbiont genus and within‐genus identity (interspecific), samples were first classified using the fragment size of a hypervariable region in domain V of the chloroplast 23S ribosomal DNA following the protocol of Santos, Gutierrez‐Rodriguez, and Coffroth (2003), detecting a given genotype at an abundance of 10–1000 cells (Santos, Gutierrez‐Rodriguez, & Coffroth, 2003). Amplicons were run on a 6.5% Long‐Ranger polyacrylamide gel (Lonza, Rockland, ME) on a LI‐COR Gene ReadIR 4200 DNA Sequencer (LI‐COR Biotechnology Division, Lincoln, NE) with positive and negative controls and allele size determined by comparison with known DNA standards. The resultant types are referred to as cp‐types herein. If duplicate symbiont cp‐types appeared across a colony (e.g., top, middle, and bottom samples), it was only used once per colony in analyses.

As microsatellite data can provide resolution of symbiont genotypes within a species, *Breviolum* cp‐type B184 symbionts (the predominant symbiont type in *O. faveolata* in the Florida Keys) were further characterized using three polymorphic microsatellite loci. Primers for loci B7Sym34, B7Sym36, and CA6.38, which have been adapted for use with *O. faveolata* (Thornhill et al., 2009), were used to amplified DNA using conditions as described in Thornhill et al. (2009). Briefly, amplification of 10 μl was performed using approximately 10 ng of DNA, 200 μM dNTP, 2.5 mM (B7Sym34/B7Sym36) or 1.5 mM (CA 6.38) MgCl_2_, 0.3 μM forward primer, 0.15 μM reverse primer, 0.15 μM fluorescent primer, *Taq* polymerase (0.5 U) and buffer (New England Biolab). Samples were initially denatured at 95°C for 2 min and then 30 cycles of 30 s denature at 95°C, 30 s annealing at 57°C, and 30 s extension at 72°C, with a final extension of 5 min at 72°C. Amplicons were run on an acrylamide gel as above and if duplicate symbiont MLGs appeared across a colony (e.g., top, middle, and bottom samples), it was only used once per colony in analyses.

### 2.3 Statistical analysis

Members of the family Symbiodiniaceae are haploid in the vegetative state (Blank, 1987; Santos & Coffroth, 2003); therefore, instances of multiple alleles per sample were interpreted as multiple symbiont genotypes within a single host individual. For microsatellite data, multilocus genotypes (MLG) were assigned to each sample, excluding samples where two alleles were detected at more than one locus. In all analyses, samples from the two experiments (2009 and 2011) were analyzed separately. A genotype accumulation curve was made using the R package poppr (v.2.9.3; Kamvar et al., 2014) with 999 iterations of random, without replacement, sampling of loci to assess the discrimination power of the 3 loci.

Chi‐squared tests of independence were used to determine whether symbiont populations in the adults and recruits differed at the cp‐ type level (inter‐and intrageneric level) using allele frequency data and were Bonferroni corrected. Alleles with less than five samples were grouped together as “others”. A clustered dendrogram was used for visualizing similarity/dissimilarity between the symbionts at the cp‐type level based on Bray–Curtis dissimilarity measures. The dendrogram was generated using R version 2.14.1 (R Core Team, 2019).

To examine the degree of differentiation of *Breviolum* populations within hosts at different sites and between *Breviolum* populations within host and recruits, we calculated Phi_PT_ (Φ_PT_ is a modified version of Wright's F_ST_ for haploid data) using an analysis of molecular variance (AMOVA) in GenAlEx 6.501 (Peakall & Smouse, 2006) of the MLG data. The option for haploid data was selected. Significant differences were tested based on Monte Carlo simulations using 9999 permutations of the data and were Bonferroni corrected (*p* < .05). To visualize the similarities/differences between groups, principal coordinates analysis (PCoA) was conducted in GenAlEx 6.501 (Peakall & Smouse, 2006). To visualize individual variation, a principal component analysis (PCA) was performed with the ade4 package (V 1.7–19; Dray & Dufour, 2007) and plotted with ggplot2 (Wickham, 2016).

## 3 RESULTS

### 3.1 Cp‐type analyses of symbionts from *O. faveolata*

**FIGURE 2:**
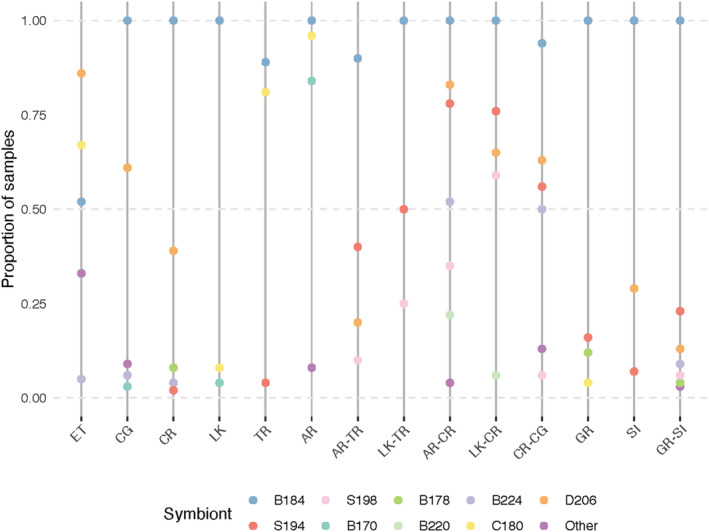
Proportion of samples with a given symbiont cp‐type in adults and recruits across reefs. Two‐letter abbreviations indicate the reef on which the adult was sampled, while four‐letter abbreviations indicate recruit source and outplant site, respectively. Site name, abbreviations, and sample sizes are given in Table 1. Cp‐type given as first letter of genus and fragment size (bp) of allele. Other—rare alleles seen in less than five samples (183, 188, 211, 215, and 230). Y‐axis values that sum to greater than 1 indicate multiple symbiont cp‐types within a single polyp. For example, for SI, 100% of the samples harbored B184 symbionts and some of these same samples also harbored 7% and 29% of S194 and D206, respectively. Zeroes (when a cp‐type was not present) are not plotted.

The frequency of multiple symbiont cp‐types within a colony was relatively high (up to 93% at the Middle Keys sites), which is consistent with observations that *O. faveolata* can host a range of symbiont cp‐types (Kemp et al., 2008; Rowan et al., 1997; Thornhill, Daniel, et al., 2006; Thornhill, LaJeunesse, et al., 2006; Toller et al., 2001). Most of the adults sampled harbored the cp‐type *Breviolum* B184, usually with other cp‐types (Figure 2). For example, at shallow sites, except for ET, cp‐type *Breviolum* B184 was found in all samples, often co‐occurring with other cp‐types (Figure 2). At the deep sites (i.e., TR and AR), symbiont diversity within adults was more equally distributed among *Breviolum* cp‐type B184 and *Cladocopium* cp‐type C180 and additionally an unidentified 170 bp allele at the AR site (Figure 2). At the ET site, *Durusdinium* cp‐type D206 was the most abundant symbiont cp‐type, found in over 85% of the adult colonies followed by *Cladocopium* C180 and *Breviolum* B184 (harbored by 67% and 52% of the colonies, respectively; Figure 2, Table A1 in Appendix 1). As at the other sites, mixtures of cp‐types were often found co‐occurring within the same colony.

Although *Breviolum* cp‐type B184 was the most common symbiont cp‐type at most sites, multiple symbiont cp‐types within an individual led to significant differences between overall symbiont compositions within adult *O. faveolata* populations in 2011 (χ^2^, *p* < .005, Bonferroni corrected; Table A2b in Appendix 2). In 2009, the overall symbiont cp‐types within adults between sites (SI vs. GR and CG vs. CR) did not differ (χ^2^, *p* > .025 Bonferroni corrected; Table A2a in Appendix 2).

DNA for recruits was limited, and often many PCR runs were required for each locus which depleted many samples. Additionally, at some sites, only a few recruits were recovered. Together, this resulted in low sample sizes at some sites. However, based on the mean of the number of cp‐types identified at each site, symbiont cp‐types within the recruits were more diverse than within the adults (mean of 6.83 vs. 4.88 symbiont cp‐types per site, respectively) (Figure 2, Table A1 in Appendix 1). As with the adults, the majority of the recruits harbored cp‐type *Breviolum* B184 along with other symbiont cp‐types (as noted by proportions adding to greater than 1 in Figure 2). For example, while 94% of the CR recruits outplanted to CG (CR‐CG recruits) harbored the common cp‐type B184, 56% of these also harbored cp‐type S194, a symbiont cp‐type that was not detected in the adults at CG, the outplant site, and rare in adults at CR (2%), the source of the planulae (Figure 2, Table A1 in Appendix 1). Cp‐types S194 and B224 (mean 77% and 64%, respectively) also co‐occurred with B184 in recruits outplanted to CR, were not detected in the adults at the parental sites (LK and AR) and rare in the adults of the outplant site (CR, 2% and 4%, respectively; Figure 2, Table A1 in Appendix 1). While 45% of the recruits outplanted to TR harbored cp‐type S194, this symbiont cp‐type was not detected in the adults at either the AR and LK sites, the source of the planulae, and was rare in the adults at TR, the outplant site (Figure 2, Table A1 in Appendix 1). Symbionts within the recruits generally differed from adults at both the parental site and the outplant site (χ^2^, *p* < .013 and .006, Bonferroni corrected; Table A2c,d in Appendix 2, respectively). The only exceptions were symbionts in GR recruits outplanted to SI, which did not differ in cp‐type from the symbionts in SI adults (χ^2^, *p* < .013, Bonferroni corrected; Table A2c in Appendix 2) and symbionts within adults at SI also did not differ from the symbionts in CR recruits outplanted to CG (χ^2^, *p* < .013, Bonferroni corrected; Table A2c in Appendix 2), although low sample sizes may contribute to the lack of statistical significance.

In contrast, the symbionts in recruits outplanted to the same site (e.g., AR‐CR vs. LK‐CR or AR‐TR vs. LK‐TR) were not significantly different, regardless of where the egg/sperm bundles were collected (χ^2^, *p* > .006, Table A2f in Appendix 2). However, there was a significant difference in the symbiont cp‐types found in the recruits between years (2009 vs. 2011) and regions (Upper vs. Middle Keys, χ^2^, *p* < .0006; Table A2e,f in Appendix 2e,f; Figure 2).

#### 3.1.1 Similarity analyses of symbionts from *O. faveolata* adults and recruits

**FIGURE 3:**
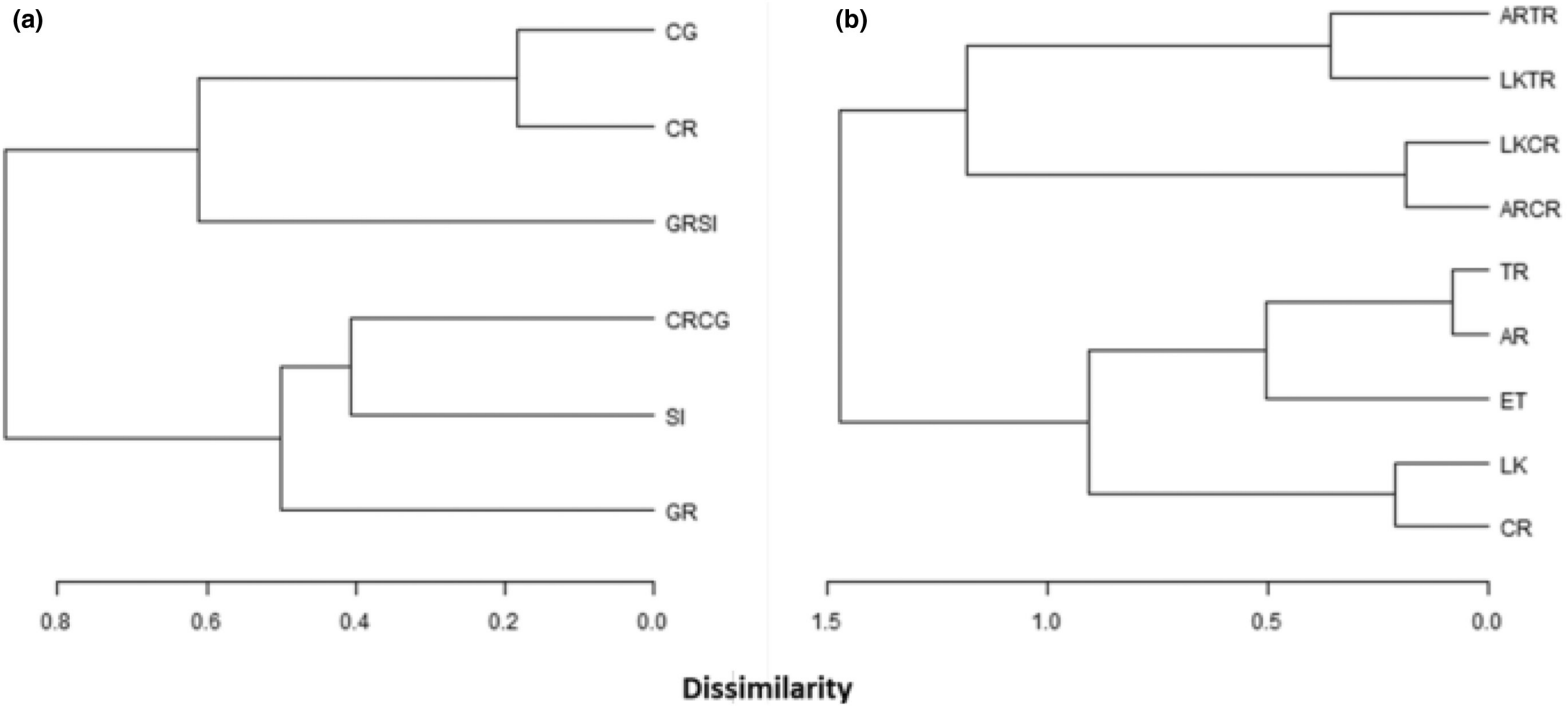
cp‐type clustered dendrogram showing similarity/dissimilarity between symbiont communities in adults and recruits at the study sites as indicated. Grouping based on Bray–Curtis similarity measures for (a) 2009 and (b) 2011. Clustered dendrogram was generated using R version 2.14.1. Reef abbreviations as in Table 1.

Cluster analysis, based on Bray–Curtis dissimilarity measures of the cp‐types, separated the symbionts isolated from adults and recruits into groupings that generally support the results of the chi‐squared analysis (Figure 3, Table A2 in Appendix 2). One exception is that the 2009 GR‐SI recruits cluster with symbionts from adults at CR and CG, in contrast to the chi‐squared analysis (Figure 3a, Table A2c in Appendix 2). In 2011, symbiont cp‐types were most similar among all recruits and distinct from symbiont cp‐types in adult colonies (Figure 3b). Symbionts within the adult colonies tend to cluster based on depth (Figure 3b), grouping adults at the deep sites of TR and AR together with the highest similarity in symbiont types (Figure 3b). Symbiont types within adult *O. faveolata* at ET were more similar to TR and AR than the rest of the sample sites (Figure 3b). Symbionts within adults sampled at the other shallow sites (CR and LK) also formed a group (Figure 3b). Symbiont cp‐types within outplanted coral recruits also group together, with the symbiont cp‐types within the recruits outplanted to the shallow Middle Keys site (CR) grouping together, the symbionts within recruits outplanted to the deeper site (TR) grouping together and neither grouping with the symbionts found within the adults at these sites.

#### 3.1.2 Symbionts in recruits prior to outplanting

Seven out of 60 recruits sampled prior to outplanting from tiles pre‐conditioned at ET harbored symbionts, revealing that a few of the newly settled corals obtained symbionts, presumably from the microflora established on the tiles during pre‐conditioning at the ET site. These pre‐outplant samples represent three recruits (out of 30 sampled) from AR larvae which harbored cp‐types B178, B184, and D206 and four recruits (out of 30 sampled) from LK larvae which harbored cp‐types B178, B184, B224, and D206. Symbionts within recruits sampled pre‐outplant were compared with symbionts within the post‐outplant recruits (i.e., recruits that had settled in the laboratory on tiles pre‐conditioned at ET or in FSW prior to outplanting). The seven recruits sampled prior to outplanting the tiles to the field were not significantly different from the symbionts found in the recruits at CR or TR after these tiles were outplanted, although low sample sizes may contribute to the lack of statistical significance (Table A2g in Appendix 2).

### 3.2 Variation within cp‐type B184 symbionts from *O. faveolata*—Microsatellite analysis

At the microsatellite level, the frequency of multiple alleles among symbionts was lower within adult colonies (ranging from 0.4% to 7.3% at a given locus; Table 2) than in the recruits (ranging from a mean of 5.0% to 39.5%). However, the frequency of multiple alleles in symbionts within adult colonies was substantially greater at ET where multiple symbiont alleles were detected in 57% of the adults with the most variability seen in locus B7Sym34.

**TABLE 2:** *Breviolum* cp‐type B184 microsatellite loci and allelic characteristics

Locus	Num. alleles	Freq. Mult. Alleles
**Adult**		
B7Sym34	15	0.073
B7Sym36	11	0.027
CA6.38	5	0.004
**Juvenile**		
B7Sym34	15	0.395
B7Sym36	5	0.390
CA6.38	6	0.050

*Note*: Three microsatellite loci were used in this study for the symbionts of *Orbicella faveolata* in both adults and recruit samples.

**FIGURE A3:**
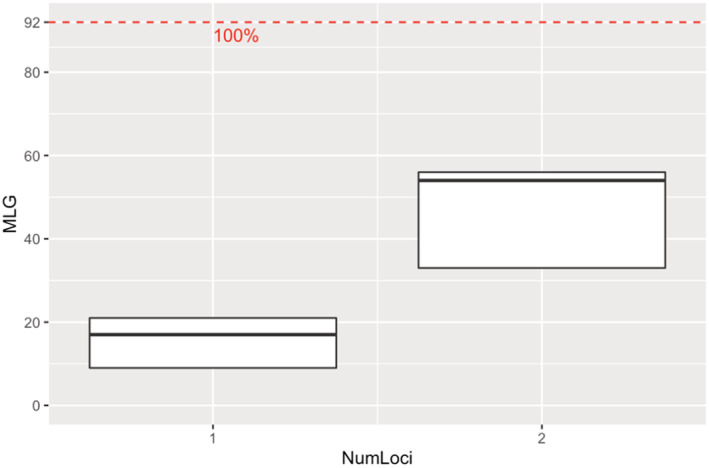
Genotype accumulation curve to assess the ability of the three microsatellite loci to discriminate between individuals.

Although only three microsatellite loci were used, these identified a total of 79 symbiont MLGs in samples collected from adults and recruits with 5–15 alleles/locus (Table 2). These three microsatellites identified 51 *Breviolum* genotypes among adults and 38 among recruits and were able to distinguish symbionts within adults from those within recruits, indicating the robustness of using these three microsatellites. A genotype accumulation curve (Figure A3 in Appendix 3) indicated that inclusion of additional microsatellites may have identified additional genotypes, but these three microsatellites enabled us to distinguish symbionts within adults from those within recruits. Of these, 28 MLGs were unique to symbionts acquired by recruits and 41 were unique to symbionts harbored by adults. Ten symbiont MLGs were shared between adults and recruits, and 11 symbiont MLGs were shared between adults on different reefs. Richness of MLGs at a site ranged from 6 to 21. There was a total of 11 recruits that shared symbiont MLGs with symbionts in adult parental and/or outplant colonies—six recruits (CR‐CG = 4; GR‐SI = 2) shared symbiont MLGs with parental and outplant adults; one recruit (LK‐CR) harbored the same symbiont MLG as an adult from the parental site, and four recruits (CR‐CG = 1; AR‐CR = 1; GR‐SI = 2) had the same symbiont MLGs as adults at the outplant site. A total of 19 recruits also shared some of these symbiont MLGs with symbionts in adults from non‐parental or non‐outplant sites (Table 3).

**TABLE 3:** Multilocus genotypes (MLG) shared between adults and recruits

	Site	Shared MLGs										Total
		1	2	3	4	5	6	7	8	9	10	MLGs#
Adults	AR	–	–	6	–	–	–	–	–	–	–	9
	CG	1	–	5	–	1	–	–	2	3	1	12
	CR	–	–	6	–	1	–	–	–	1	2	8
	ET	–	–	2	1	–	–	–	–	–	2	11
	GR	–	–	3	–	–	2	–	–	–	1	9
	LK	–	1	45	–	–	–	1	–	7	–	21
	SI	1	–	2	–	–	1	–	–	1	–	10
	TR	–	–	17	–	1	–	–	–	2	–	6
Recruits	GR to SI	–	1	**2**	2	–	–	3	–	**2**	–	**17**
	CR to CG	–	–	**1**	–	**1**	–	–	**1**	**1**	**1**	**7**
	AR TO CR	1	–	–	–	–	–	–	–	**1**	–	10
	LK TO CR	1	–	–	–	–	1	**1**	1	–	–	11
	AR to TN	–	–	–	–	–	–	–	–	–	–	1*
	LK to TN	–	–	–	–	–	–	–	–	–	–	*

*Note*: Numbers indicate number of times that a MLG was found at a site. Numbers above columns are arbitrary names signifying the MLG designation that was shared between adults and recruits. Total MLGs are the total number of different genotypes found at the site. Yellow—MLG shared with adult at both parent and outplant site; blue—MLG shared with adult at parent site only; green—MLG shared with adult at the outplant site only; white—MLG shared with adults at other sites, but not parent or outplant site. *MLGs could not be assigned due to numerous multiple alleles. Reef abbreviations as in Table 1.

**FIGURE 4:**
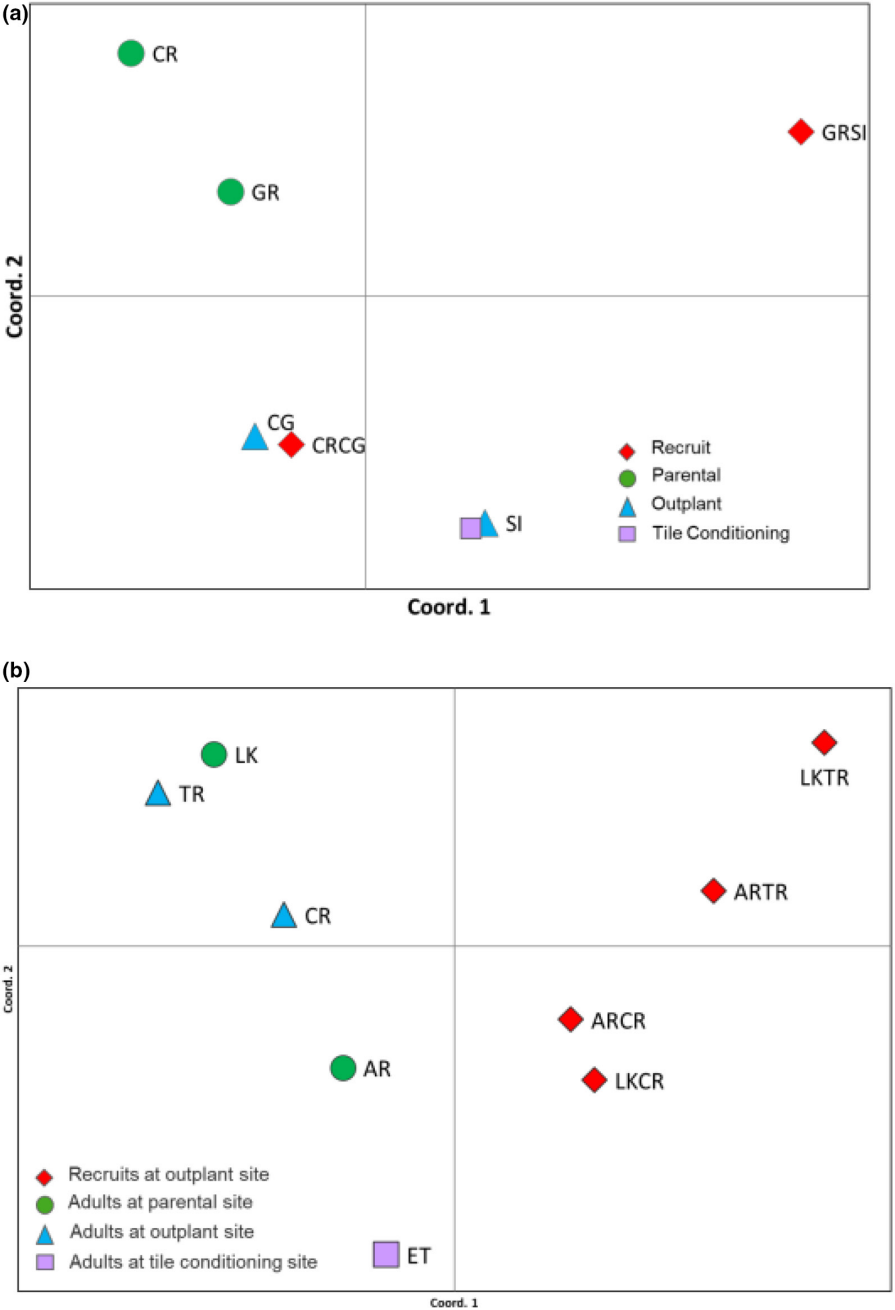
Symbiont populations visualized through principal coordinates analysis (PCoA) of MLG data (a) symbionts in adults and recruits from 2009 data (b) symbionts in adults and recruits from 2011 data. Reef abbreviations as in Table 1.

**FIGURE A6:**
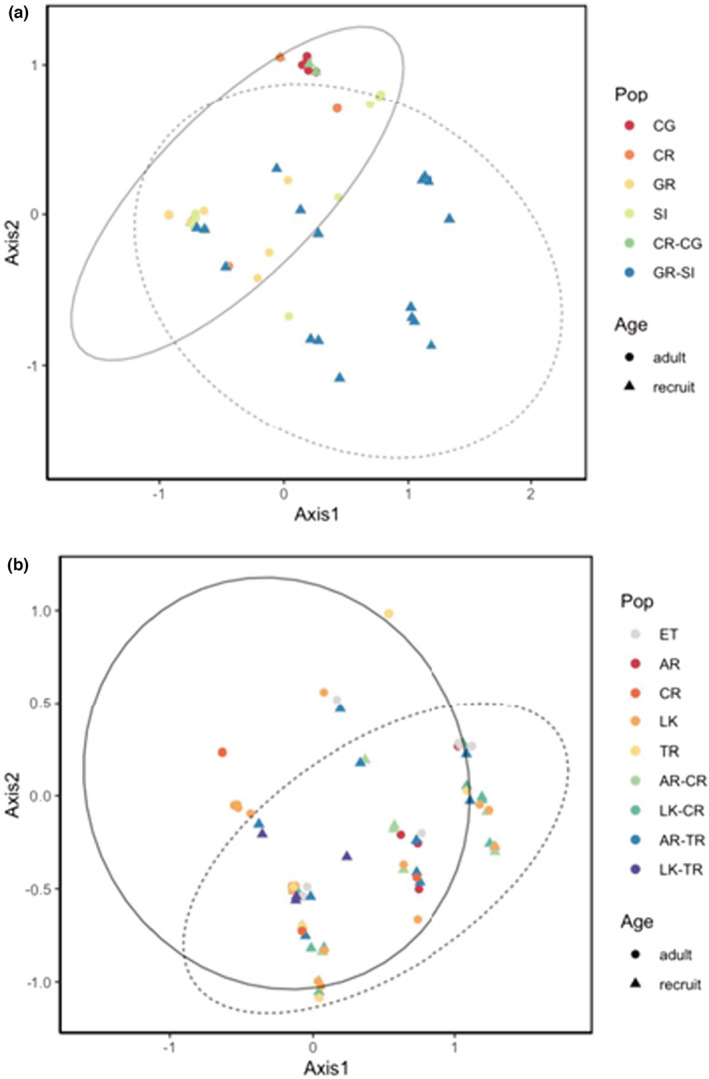
PCA showing individual variation in results for recruits and adults, (a) 2009 (b) 2011. Solid ellipse—adults; dotted ellipse—recruits. Reef abbreviations as in Table 1.

AMOVA of microsatellite allele data revealed that in 2009 symbionts acquired by recruits reared from egg‐sperm bundles collected at CR (parental site) and outplanted to CG (CR‐CG recruits) were not significantly different from symbionts in adults from CG, CR, GR, or SI (Φ_PT_ given in Table A4 in Appendix 4), although low sample size may contribute to this. Symbionts in adults at GR and CG also did not differ significantly (Table A4 in Appendix 4, Φ_PT_—0.1564, AMOVA, *p* < .0033, Bonferroni corrected). However, symbionts in CR‐CG recruits were significantly different from symbionts within GR‐SI recruits (Φ_PT_—0.26000, AMOVA, *p* < .0033, Bonferroni corrected). Furthermore, symbionts in recruits outplanted to SI (GR‐SI recruits) were not similar to symbionts in adults at either the parental site (GR) or the outplant site (SI; see Table A4 in Appendix 4). These distinctions are evident in the PCoA plot where symbionts within GR‐SI recruits were clearly distinguished from symbionts within both parental site and outplant site adults (first and second axes explaining 51.5% and 24.5% of variation, respectively), while symbionts within CR‐CG recruits grouped with symbionts within outplant‐site adults, although not with symbionts in adults at the parental reef as the AMOVA indicated (Figure 4a and Figure A6a in Appendix 6). Symbionts in GR‐SI recruits were distinct from parental site adults' symbionts (Figure 4a).

In 2011, symbionts in recruits outplanted to CR and TR (AR‐CR, LK‐CR, AR‐TR, and LK‐TR recruits) were similar to each other but differed from symbionts in adults at the parental (LK and AR) and outplant (CR and TR) sites as well as ET, the site where settlement tiles were pre‐conditioned (Table A5 in Appendix 5, AMOVA, *p* < .0014, Bonferroni corrected). Symbionts in adults from the Middle Keys site TR were similar to symbionts within adults at LK sites (Table A5 in Appendix 5, AMOVA, *p* = .1403). Symbionts in all other adults differed between sites (Table A5 in Appendix 5, AMOVA, *p* < .0014, Bonferroni corrected). A PCoA plot corresponded with the similarities seen in the AMOVA results, with 52.6% and 20.8% of variation explained by the first and second axes, respectively (Figure 4b), while a PCA demonstrated individual variation (Figure A6b in Appendix 6).

## 4 DISCUSSION

### 4.1 Species‐level diversity of symbionts in adults and newly settled recruits

Symbiont diversity in *O. faveolata* recruits was both high and more variable than in adult populations, with recruits acquiring multiple Symbiodiniaceae phylotypes (at both the genus and species level), often simultaneously, within the first few months. This flexibility in initial uptake by coral recruits relative to adults has been demonstrated in a number of coral species (e.g., Ali et al., 2019; Chan et al., 2019; Coffroth et al., 2001; Gómez‐Cabrera et al., 2008; Poland et al., 2013), and several, non‐exclusive, hypotheses have been proposed to explain this pattern. The tendency for newly settled corals to harbor a distinct and/or more diverse array of symbiont types than their adult conspecifics may be the byproduct of an undeveloped immune system in recruits (Frank et al., 1997; Nozawa & Loya, 2005; Puill‐Stephan et al., 2012) or the suppression of the host immune response by the symbionts (Jacobovitz et al., 2021; Mansfield & Gilmore, 2019; Schnitzler & Weis, 2010; Voolstra et al., 2009; Yoshioka et al., 2022). Then, as recruits age and presumably as the immune system develops, recruits are less likely to take up new symbiont genotypes (McIlroy & Coffroth, 2017). Differences in the microhabitat across the coral colony can also affect symbiont species distributions in both adults and recruits (Abrego et al., 2009a; Rowan et al., 1997) and may contribute to differences between recruits and adults. Dramatic changes in environmental conditions for *in hospite* symbionts that occur as corals age and grow, particularly during early growth stages, may lead to fast turnover in competitively dominant symbiont genotypes (Lecointe et al., 2016; Nitschke et al., 2015).

Finding high diversity and multiple symbiont types within coral recruits is not new, and as with other studies, this high symbiont diversity within new recruits recorded here may benefit these *O. faveolata* recruits that likely settle far from their natal reef with possibly different environmental conditions (light, nutrients, etc.; Abrego et al., 2009b; Ali et al., 2019; Puill‐Stephan et al., 2012; Poland & Coffroth, 2017, 2019). Additionally, harboring multiple different symbiont types may enable new recruits to respond to the ontogenetic changes in energetic requirements as the coral develops. Despite the potential benefit of symbiont diversity and flexibility in recruits, growth and survival of coral recruits in the first months to years varies with symbiont genotype and some studies have demonstrated that harboring multiple symbiont types may not be beneficial (Little et al., 2004; McIlroy et al., 2016; Poland & Coffroth, 2019). For example, Poland and Coffroth (2019) showed that within the octocoral *B. asbestinum*, recruits that initially harbored non‐*Breviolum* spp. or a mixture of *Breviolum* and other genera, had slower growth and greater mortality. Thus, the high symbiont diversity and multiple symbiont genotypes seen within *O. faveolata* recruits may not be beneficial in all cases. The mechanisms that determine symbiont community diversity and the consequences of this winnowing process on recruit fitness remain topics in need of additional research.

Our study corroborates other studies (Baums et al., 2010; Kemp et al., 2015; LaJeunesse, 2002; Thornhill et al., 2009) that found that *O. faveolata* adults in the Florida Keys are dominated by symbionts within the genus *Breviolum* and here show that newly settled recruits are also dominated by *Breviolum* symbionts (Figure 2). Host specificity at the level of intrageneric/species (i.e., ITS2‐type), as demonstrated here, is well known (e.g., LaJeunesse, 2002; LaJeunesse et al., 2010; Rodriguez‐Lanetty et al., 2006; Thornhill et al., 2014), which is perhaps unsurprising given the risk of associating with sub‐optimal or potential negative interactions among symbionts. Thus, although there is diversity in the Symbiodiniaceae of newly settled recruits, there is also heritability across generations, even when symbionts are acquired horizontally, accounting for 29% of the symbiont community of *Acropora tenuis* juveniles in the first month (Quigley, Willis, & Bay, 2017). The symbiont genus/species that comes to dominate in the adult can often be detected in early ontogeny (Poland & Coffroth, 2017). This may factor into the winnowing in symbiont types, which subsequently occurs and leads to the establishment of the adult symbiont community over months to years (Abrego et al., 2009a; Chan et al., 2019; Little et al., 2004; Poland & Coffroth, 2017). Almost 100% of both *O. faveolata* adults and recruits harbored *Breviolum* B1/B184 (ITS2‐type/cp‐type), a frequency that points to an intergenerational, that is, genetic, predisposition for this symbiosis across the Florida Keys at the intrageneric/species (i.e., ITS2‐type/cp‐type) level. Unfortunately, high mortality of the recruits in the field limited our observations to the first 3 months, so that we were not able to monitor how symbiont diversity in *O. faveolata* recruits would have been reduced over time, as has been reported for other corals.

### 4.2 Population‐level diversity of symbionts in adults and newly settled recruits

For corals, population‐level assessments of symbiont associations can be confounded by the generic diversity of symbionts within a coral host. Variation in diversity based on cp‐types within the symbiont communities in the recruits could account for the similarities and differences seen between symbionts harbored by adults and recruits at the level of between symbiont genera and species. However, at a finer level of resolution, the three *Breviolum* microsatellites used in this study were effective at distinguishing the adult populations, demonstrating that the overall symbiont assemblages in adults on one reef tended to differ from those in adults on other reefs (Tables A4 and A5 in Appendixes 4 and 5). This suggests that endemism is common among symbiont populations of *O. faveolata* on a reef scale, which may be due to local selection upon and/or limited dispersal by symbionts (Howells et al., 2009; Kirk et al., 2005, 2009; Santos, Gutiérrez‐Rodríguez, et al., 2003; Thornhill et al., 2009). Yet, there were exceptions to this where symbionts in adults at GR did not differ from symbionts in adults from SI or CR and symbiont populations in adults on TR did not differ from those in adults on LK (Tables A4 and A5 in Appendixes 4 and 5, Figure 4). Although proximity might account for the similarity between symbionts in adults at GR and SI (~14 km apart), the distances between reefs GR and CR and between TR and LK are greater (~46 and 35 km apart, respectively). It is possible that these populations are highly mixed and lack fine‐scale structure. Connectivity between different regions may be aided by the northward flow of the Florida Current or local eddies and wind‐driven counter currents in some cases (Drury et al., 2018; Lee et al., 1994; Pitts, 1994). Although increasing the number of loci would not necessarily change our overall findings (Björklund & Bergek, 2009), additional loci and/or larger sample sizes could possibly better distinguish symbionts between these groups and provide a better understanding of the population structure of symbionts in these areas (Figure A3 in Appendix 3).

In contrast to symbiont communities within the adults, few studies have examined specificity at the population level (but see Andras et al., 2011; Poland & Coffroth, 2017). We found that symbiont assemblages within recruits within a region were similar overall at both the cp‐type and MLG levels (Figure 3, Tables A2f and A5 in Appendixes 2 and 5). Recruits deployed at a particular reef or region tended to group together based on similarity of symbiont types regardless of the source of those larvae, although additional loci and/or larger sample sizes could possibly better distinguish symbionts between these groups. While symbiont genotypes taken up by recruits in the Middle Keys did not differ between shallow (CR) and deep (TR) outplant sites (Figure 3b, Tables A2f and A5 in Appendixes 2 and 5), on a more regional scale, symbionts in Upper Keys recruits (GR‐SI) differed from symbionts genotypes found in Middle Keys recruits (CR‐CG) (Figure 3a, Tables A2e and A4 in Appendixes 2 and 4). These findings suggest that the light environment (depth) has little influence on the symbiont genotypes that first enter symbiosis with newly settled recruits, and instead that the pool of symbiont genotypes available for uptake is influenced by other environmental conditions, physical distance, and perhaps regional scale currents.

Our study suggests parental effects of *O. faveolata* do not limit which populations of *Breviolum* B1/B184 are acquired by newly settled recruits. A comparison of microsatellite MLGs in adults and recruits indicates that, in most cases, recruits harbor a different set of B184 symbionts compared with the communities within adult *O. faveolata* with only 10 of the 79 MLG shared between symbionts within adults and recruits (Table 3). This lack of concordance of genotypes between symbionts in recruits and adults contrasts with reports of parental genetic influences in symbiont acquisition in some hosts with horizontal transmission. For example, *Briareum asbestinum* recruits shared unique microsatellite alleles with parental populations regardless of outplant location (Poland & Coffroth, 2017). Overall, the comparisons between symbiont types in adults and recruits of *O. faveolata* indicate that parental effects may have limited influence on the MLGs of symbionts that the newly settled host initially acquires, despite specificity at the symbiont genus and/or species level. These finding are important as they indicate that the factors that influence the initial symbiont population genotypes acquired may vary among host species and allow for uptake of potentially locally adapted genotypes.

### 4.3 Potential sources of symbiont diversity

To understand what might lead to the difference in symbiont MLGs harbored by adults and recruits, we need to consider the potential sources of symbionts for newly settled coral. Symbiodiniaceae are released into the reef environment by adult corals (Koike et al., 2007; Muscatine & Pool, 1979; Stimson & Kinzie, 1991) and have been recovered from the water column, macroalgae, and sediments (e.g., Adams et al., 2009; Coffroth et al., 2006; Littman et al., 2008; Manning & Gates, 2008; Porto et al., 2008; Takabayashi et al., 2012). Numerous studies have demonstrated that newly settled recruits do acquire symbionts from the water column, sediments and, in lab settings, from nearby coral colonies (Ali et al., 2019; Coffroth et al., 2006; Cumbo et al., 2013; Nitschke et al., 2015; Quigley et al., 2019; Sweet, 2014; Williamson et al., 2021). Since outplant‐site adults and recruits are on the same reef and thus in similar environments, we might predict that the symbionts within these two life stages would be similar. Our results suggest that the adult hosts at the site might not be the major source of symbionts, which is similar to other studies where the predominant symbionts in surrounding corals and other cnidarian hosts often differed from those found in nearby recruits (Abrego et al., 2009b; Ali et al., 2019; Andras et al., 2011; Chan et al., 2019; Gómez‐Cabrera et al., 2008; Little et al., 2004; Mellas et al., 2014; Poland et al., 2013; Thornhill, Daniel, et al., 2006; Thornhill, LaJeunesse, et al., 2006; Yamashita et al., 2013). Even in lab settings, where coral colonies are seemingly the only source of symbionts, recruits can harbor symbiont types that differ from the adult source colony (Ali et al., 2019; Williamson et al., 2021).

Temporal and spatial variation in local environmental symbiont pools have been posited to explain the variation in symbiont types harbored by recruits from different locations as well as the differences in symbiont types harbored by recruits and adults at the same location (Andras et al., 2011; Cumbo et al., 2013; Howells et al., 2013; Manning & Gates, 2008; Quigley, Bay, & Willis, 2017; Sweet, 2014; Thornhill et al., 2017). We found that, in most cases, the MLGs of *Breviolum* B184 symbionts in recruits differed from symbionts in adults at the outplant site suggesting that recruits may be sampling a different symbiont pool (Tables A4 and A5 in Appendixes 4 and 5). These data are consistent with the hypothesis that the local symbiont pool changes temporally, possibly reflecting local environmental changes. Thus, when the adult symbiosis was established, the symbiont MLGs present in the environment may have differed from MLGs in the current ambient symbiont pool. McIlroy and Coffroth (2017) demonstrated that over time, newly settled *O. faveolata* recruits are less likely to acquire new symbionts. Although recruits harbor the *Breviolum* B184 cp‐type that is common in local adults, it is unlikely that recruits will switch to the adult MLGs. Alternatively, if only the adult MLGs are suitable for that locale, it is possible that recruits without the adult MLGs do not survive. Monitoring recruits in the field for longer periods of time would help to address this. Furthermore, adults MLG had higher within than among population variance (2009: 73% vs. 27%; 2011: 77% vs. 23%, within vs. among populations; AMOVA). Similarly, for *Acropora millepora* on the Great Barrier Reef, variation within sites explained 70%–86% of the total variation (Howells et al., 2013). This suggests that at the MLG level, a range of suitable genotypes have been present over time. Adults at a given site likely represent many cohorts (i.e., recruitment events), and diversity among adults at a site suggests that temporal change in local symbiont pools is possible. Changes in these environmental symbiont pools have been attributed to physical disturbance (flooding and hurricanes), which could redistribute benthic dwelling symbionts (Howells et al., 2013), genetic drift, or adaptations to a changing environment (Andras et al., 2011; Howells et al., 2013). Studies have demonstrated that within Symbiodiniaceae species, different strains display different physiologies, including thermal tolerance (Bayliss et al., 2019; Beltrán et al., 2021; Diaz‐Almeyda et al., 2017; Howells et al., 2012; Klueter et al., 2017; Parkinson & Baums, 2014; Pelosi et al., 2021). In addition to standing variation within symbiont species, studies suggest that mutation rates among these protists are high (van Oppen et al., 2011) and laboratory studies have demonstrated the potential for rapid thermal adaption (Buerger et al., 2020; Chakravarti et al., 2017). If symbiont pools are changing over time, the next step will be to determine whether this change in the local symbiont pools reflects adaptations in symbionts in response to a changing climate. If so, this may suggest hope for a new generation of more resilient corals.

## AUTHOR CONTRIBUTIONS

**Mary Alice Coffroth**: involved in conceptualization (lead); funding acquisition; formal analysis (equal); investigation (equal); methodology (equal); project administration (lead); resources (lead); supervision (lead); visualization (equal); writing—original (lead); and writing—review and editing (equal). **Noel J. Leigh**: involved in conceptualization (supporting); formal analysis (supporting); investigation (equal); methodology (equal); visualization (equal); writing—original (supporting); and writing—review and editing (equal). **Shelby E. McIlroy**: involved in investigation (equal); methodology (equal); formal analysis (equal); supervision (supporting) visualization (equal); and writing—review and editing (equal). **Margaret W. Miller**: involved in funding acquisition; methodology (equal); project administration; (supporting); and writing—review and editing (equal). **H. David Sheets**: involved in formal analysis (equal); software (lead); visualization (equal); and writing—review and editing (equal).

## CONFLICT OF INTEREST

On behalf of all authors, the corresponding author states that there is no conflict of interest.

## Data Availability

The data that support these findings have been submitted to the BCO‐DMO database and can be found at https://www.bco-dmo.org/search?mefibs-form-search-es-multi-header-keywords=coffroth&mefibs-form-search-es-multi-header-mefibs_block_id=search_es_multi_header.

## APPENDIX 1

**TABLE A1:** cp‐type allele frequencies in adults and recruits of *Orbicella faveolata*

Allele size (bp)	2009						2011								
	GR	SI	GR‐SI	CR	CG	CR‐CG	AR	CR	AR‐CR	LK‐CR	LK	LK‐TR	AR‐TR	TR	ET
B184	**1**	**1.00**	**1.00**	**1.00**	**1.00**	**0.94**	**1.00**	**1.00**	**1.00**	**1.00**	**1.00**	**1.00**	**0.90**	**0.89**	0.52
S194	0.16	0.07	0.23	0.02	–	0.56	–	0.02	0.78	0.76	–	0.50	0.40	0.04	–
S198	–	–	0.06	–	–	0.06	–	–	0.35	0.59	–	0.25	0.10	–	–
B170	0.16	–	–	–	0.03	–	0.84	–	–	–	0.04	–	–	–	–
B178	0.12	–	0.04	0.08	0.03	–	–	0.08	0.35	0.76	–	0.25	0.10	–	–
B220	–	–	–	–	–	–	–	–	0.22	0.06	–	–	–	–	–
B224	–	–	0.09	0.04	0.06	0.50	–	0.04	0.52	0.76	–	–	0.10	0.04	0.05
C180	0.04	–	–	0.04	–	–	0.96	0.04	–	–	0.08	–	–	0.81	0.67
D206	–	0.29	0.13	0.39	0.61	0.63	–	0.39	0.83	0.65	0.04	0.25	0.20	–	**0.86**
Other^a^	0.04	–	0.03	0.02	0.09	0.13	0.08	0.02	0.04	–	–	–	0.10	–	0.33
*n*	26	14	109	35	53	16	29	35	23	16	50	4	10	27	22
# Alleles	6	3	7	7	6	7	4	7	8	7	4	5	7	4	5
*n* Multi. Alleles	6	4	44	23	19	12	27	23	20	5	9	3	3	19	17
Freq. of Multi. Alleles	0.23	0.29	0.40	0.66	0.36	0.75	0.93	0.66	0.87	0.31	0.18	0.75	0.30	0.70	0.77

*Note*: Cp‐type given as first letter of genus and fragment size (bp) of allele. Other—rare alleles seen in less than five samples. n is the number of samples that were screened for sequence variation within a hypervariable region of domain V of the 23s rDNA chloroplast gene. # Alleles are the number of different alleles detected at a given location. *n* Multi. Alleles are the number of samples that contained multiple alleles. White columns are the data for symbionts in coral adults. Gray columns are the data for symbionts in coral recruits. Dominate type highlighted in bold. Reef abbreviations as in Table 1.

^a^ Other = 183, 188, 211, 215, 230.

## APPENDIX 2

**TABLE A2:** Chi‐squared comparisons of cp‐type data

a				
Adult comparisons between sites—2009s		χ^2^	df	*p*
GR	SI	0.554	2	.457
CR	CG	1.352	2	.509

b				
Adult comparisons between sites—2011		χ^2^	df	*p*
TR	CR	44.40	3	**1.24E‐09**
AR	CR	69.179	4	**6.40E‐15**
AR	TR	16.715	3	**8.08E‐04**
LK	CR	15.431	3	**1.48E‐03**
LK	AR	36.319	3	**16.41E‐08**
LK	TR	21.206	2	**2.48E‐05**
ET	CR	42.33	4	**1.43E‐08**
ET	LK	50.382	4	**3.01E‐10**
ET	TR	31.878	4	**2.03E‐06**
ET	AR	38.127	5	**3.55E‐07**

c				
Adult colonies	Recruits from parental site to outplant site—2009	χ^2^	df	*p*
SI	GR‐SI	7.042	5	0.22
GR	GR‐SI	22.956	5	**3.44E‐04**
CG	CR‐CG	27.093	4	**1.90E‐05**
CR	CR‐CG	25.433	4	**4.12E‐05**
SI	CR‐CG	11.31	4	2.33E‐02
GR	CR‐CG	25.01	4	**5.01E‐05**
CG	GR‐SI	35.13	6	**4.07E‐06**
CR	GR‐SI	26.42	6	**1.86E‐04**

d				
Adult colonies	Recruits from parental site to outplant site‐ 2011	χ^2^	df	*p*
AR	AR‐CR	114.68	9	**1.63E‐20**
CR	AR‐CR	60.007	7	**1.50E‐10**
AR	AR‐TR	41.031	3	**6.44E‐09**
TR	AR‐TR	26.579	2	**1.69E‐06**
LK	LK‐CR	73.709	6	**7.08E‐14**
CR	LK‐CR	51.766	6	**2.08E‐09**
LK	LK‐TR	8.689	1	**0.003**
TR	LK‐TR	20.470	2	**3.589E‐05**

e				
Recruits: Parental to outplant 2009	Recruits: Parental to outplant	χ^2^	df	*p*
GR‐SI	CR‐CG	19.205	5	**.0012**

f				
Recruits: Parental to outplant 2011	Recruits: Parental to outplant	χ^2^	df	*p*
AR‐CR	LK‐CR	8.881	7	0.261
AR‐TR	LK‐TR	0.021	1	0.885
AR‐CR	AR‐TR	7.532	7	0.376
LK‐CR	LK‐TR	3.700	6	0.717
AR‐TR	LK‐CR	8.738	6	0.189
AR‐CR	LK‐TR	3.491	7	0.836
All recruits at TR	All recruits at CR	11.850	7	0.106
2009 recruits	2011 recruits	56.683	7	**6.91E‐10**
All Middle Keys recruits	All Upper Keys recruits	60.534	7	**1.18E‐10**

g				
Recruits sampled pre‐outplant	Recruits sampled Post‐outplant	χ^2^	df	*p*
All Recruits sampled pre‐outplant	Recruits outplanted to CR	8.02	6	0.237
All recruits sampled pre‐outplant	Recruits outplanted to TR	3.080	2	0.214
All recruits sampled pre‐outplant	All recruits sampled post‐outplant	6.87	6	0.333

*Note*: Chi‐squared comparisons between the symbionts in the adults, recruits, and pre‐outplant recruits at the various sites. (a) Adult between sites, 2009 (Bonferroni correction *p* < .025); (b) adult between sites, 2011 (Bonferroni correction *p* < .005); (c) recruit vs. adult, 2009 (Bonferroni correction *p* < .013); (d) recruit vs. adult, 2011 (Bonferroni correction *p* < .006); (e) recruits between sites, 2009; (f) recruits between sites, 2011 (Bonferroni correction *p* < .006); (g) recruits sampled pre‐outplant vs. post‐outplant (Bonferroni correction *p* < .017). Significant differences in bold.

## APPENDIX 4

**TABLE A4:** Phi_PT_ (Φ_PT_) of 2009 symbiont populations using *Breviolum* microsatellite loci

CG (12)	CR (8)	GR (9)	SI (10)	GR‐SI (17)	CR‐CG (7)	
–	**0.0030**	**0.0008**	**0.0007**	**0.0001**	0.3867	CG
0.1249	–	0.0178	**0.0001**	**0.0001**	0.0379	CR
0.1564	0.0756	–	0.0138	**0.0001**	0.0261	GR
0.1636	0.2624	0.1341	–	**0.0001**	0.0882	SI
0.3018	0.3676	0.3163	0.2232	–	**0.0002**	GR‐SI
0.0000	0.1191	0.0904	0.0879	0.2600	–	CR‐CG

*Note*: Pairwise Φ_PT_ values for *Breviolum* populations in *O. faveolata* adults and recruits sampled in 2009 at the four sites in the Florida Keys dataset (lower diagonal), significance found in upper diagonal. Significant comparisons in bold, *p* < .0033, Bonferroni corrected. Φ_PT_ were generated using GenAlex 6.501. Parenthetical values are no. of MLGs. Reef abbreviations and sample numbers given in Table 1.

## APPENDIX 5

**TABLE A5:** Phi_PT_ (Φ_PT_) of 2011 symbiont populations using *Breviolum* microsatellite loci

AR (9)	CR (8)	ET (11)	LK (21)	TR (6)	AR‐CR (10)	LK‐CR (11)	AR‐TR (1*)	LK‐TR (*)	
–	**0.0003**	**0.0011**	**0.0001**	**0.0003**	**0.0008**	**0.0001**	**0.0001**	**0.0001**	AR
0.1594	–	**0.0001**	**0.0001**	**0.0004**	**0.0001**	**0.0001**	**0.0001**	**0.0002**	CR
0.1080	0.2945	–	**0.0001**	**0.0001**	**0.0001**	**0.0003**	**0.0001**	**0.0001**	ET
0.1696	0.1611	0.2934	–	0.4061	**0.0001**	**0.0001**	**0.0001**	**0.0002**	LK
0.1575	0.1932	0.3034	0.0000	–	**0.0001**	**0.0001**	**0.0001**	**0.0001**	TR
0.1593	0.2280	0.2243	0.2738	0.3320	–	0.1403	0.0087	0.0038	AR‐CR
0.1961	0.2675	0.1936	0.3277	0.3720	0.0415	–	0.0036	0.0042	LK‐CR
0.2514	0.3606	0.3143	0.3945	0.4402	0.1295	0.1433	–	0.1371	AR‐TR
0.4460	0.5327	0.5206	0.5043	0.6251	0.2435	0.2522	0.0808	–	LK‐TR

*Note*: Pairwise Φ_PT_ values for *Breviolum* populations in *O. faveolata* adults and recruits sampled in 2011 at the five sites in the Florida Keys dataset (lower diagonal), significance found in upper diagonal. Significant comparisons in bold, *p* < .0014, Bonferroni corrected. Φ_PT_ were generated using GenAlex 6.501. Parenthetical values are no. of MLGs. *MLGs could not be assigned due to numerous multiple alleles. Reef abbreviations and sample numbers are given in Table 1.
